# The effect of remimazolam versus conventional anesthesia on postoperative delirium in frail patients: a prospective, controlled cohort study

**DOI:** 10.3389/fmed.2025.1684779

**Published:** 2025-12-09

**Authors:** Yanjie Yang, Zhangnan Sun, Jiangli Wu, Lining Huang, Shuxian Ma

**Affiliations:** Department of Anesthesiology, The Second Hospital of Hebei Medical University, Shijiazhuang, China

**Keywords:** remimazolam, postoperative delirium, frail elderly patients, general anesthesia, prospective cohort study

## Abstract

**Background:**

Frail elderly patients are at high risk for postoperative delirium (POD), a serious complication associated with poor outcomes. The choice of anesthetic agent may represent a modifiable risk factor. This study aimed to compare the effect of anesthesia involving remimazolam versus conventional general anesthesia without remimazolam on the incidence of POD in this vulnerable population.

**Methods:**

We enrolled frail elderly patients (defined as Clinical Frailty Scale ≥ 5) scheduled for elective non-cardiac surgery. Patients received either anesthesia involving remimazolam (R group, *n* = 301) or conventional general anesthesia without remimazolam (C group, *n* = 305), based on the attending anesthesiologist’s clinical decision. The primary outcome was the incidence of POD within the first three postoperative days, assessed by trained, blinded researchers using the 3-min Confusion Assessment Method (3D-CAM). Secondary outcomes included postoperative recovery quality (agitation, sleep, pain) and intraoperative safety parameters.

**Results:**

Between June 2024 and June 2025, a total of 606 patients were enrolled and analyzed (R group: *n* = 301; C group: *n* = 305). The overall incidence of POD was significantly lower in the R group (119/301, 39.5%) compared to the C group (143/305, 46.9%) (Relative Risk 0.84; 95% CI 0.71–0.99; *P* = 0.038). The remimazolam group also experienced less emergence agitation and, by postoperative day 3, reported significantly better sleep quality and lower pain scores (*P* < 0.01). A multivariate logistic regression identified the use of remimazolam as an independent protective factor against POD (Adjusted Odds Ratio 0.68, 95% CI 0.47–0.98, *P* = 0.041), while advanced age and longer anesthesia duration were independent risk factors.

**Conclusion:**

In this cohort of frail elderly patients, the use of remimazolam for general anesthesia was associated with a lower incidence of postoperative delirium compared to conventional anesthesia. This choice represents a promising, modifiable strategy for improving neurological outcomes in this high-risk group.

## Introduction

The convergence of a globally aging population and advancing surgical techniques has led to a substantial increase in the number of elderly patients undergoing surgery, as evidenced by the fact that the growth rate of surgical procedures in older individuals has surpassed the rate of population aging itself over the past two decades (1). A significant proportion of these patients present with frailty, a clinical syndrome defined by a diminished physiological reserve, reduced homeostatic capacity, and heightened vulnerability to stressors such as surgery and anesthesia (2, 3). Frailty is a powerful independent predictor of adverse postoperative outcomes, including increased mortality, prolonged hospital stays, functional decline, and major complications (4). Among these, postoperative delirium (POD) stands out as one of the most common and devastating neurological events affecting the elderly.

Postoperative delirium is an acute and fluctuating disturbance of attention, awareness, and cognition that develops in the perioperative period (5). Its incidence varies widely depending on the patient population and type of surgery, but it can affect over 50% of elderly patients after major procedures, with frail individuals being at particularly high risk (6–8). The consequences of POD are severe and far-reaching, leading to increased healthcare costs (9), a higher likelihood of long-term cognitive impairment resembling dementia (10), loss of independence (11), and a two- to three-fold increase in mortality (12, 13). Given these profound impacts, identifying modifiable risk factors and implementing preventative strategies are paramount in perioperative care.

The choice of anesthetic agent is a key modifiable factor that may influence the risk of POD. Anesthetic drugs can directly affect cerebral hemodynamics, neurotransmission (particularly GABAergic and cholinergic pathways), and inflammatory responses, all of which are implicated in the complex pathophysiology of delirium (14). Traditional agents, such as propofol, while widely used, have been contentiously linked to POD, with debates ongoing about their relative safety in vulnerable populations (15). Remimazolam tosylate, an ultra-short-acting benzodiazepine, has emerged as a promising alternative for general anesthesia (16). It is a soft drug that acts as a GABA-A receptor agonist, but its unique ester linkage allows it to be rapidly and predictably metabolized by non-specific tissue esterases, a process independent of organ function (17). This results in minimal accumulation, a more stable hemodynamic profile, and a rapid, clear-headed recovery. These properties suggest remimazolam could be beneficial in reducing POD incidence (18), but robust evidence specifically within the frail elderly population remains limited. While some randomized trials and meta-analyses have shown mixed or neutral results regarding remimazolam’s effect on POD (19, 20), others in specific high-risk populations like frail patients have suggested a benefit (21). Therefore, robust, prospective data from large, well-defined cohorts of frail patients are still critically needed to clarify this relationship and enhance the generalizability of these findings.

Therefore, our primary objective was to prospectively compare the incidence of postoperative delirium in frail elderly patients receiving anesthesia involving remimazolam versus conventional general anesthesia without remimazolam. Our secondary objectives were to compare postoperative recovery quality (agitation, sleep, pain) and intraoperative safety profiles between the two groups, and to perform a multivariate analysis to identify independent risk and protective factors for the development of POD in this specific cohort.

## Materials and methods

### Trial design

This study was a prospective, two-group, parallel, non-randomized, controlled cohort study. Patients were allocated to one of two groups based on the primary anesthetic maintenance agent selected by the clinical team. The allocation ratio was not pre-specified and depended on clinical practice patterns during the enrollment period. The study protocol received Ethics Approval from the ethics committee of The Second Hospital of Hebei Medical University (Approval No. 20242388), and the study was conducted in accordance with the principles of the Declaration of Helsinki. No important changes were made to the study methods after commencement.

### Participants

We enrolled participants from June 2024 to June 2025 at The Second Hospital of Hebei Medical University, a university-affiliated Class-A tertiary hospital in Shijiazhuang, China. Eligibility criteria for participants were as follows: (1) age ≥ 50 years; (2) preoperatively assessed as frail, defined by a Clinical Frailty Scale (CFS) score of 5 or higher (3); and (3) scheduled for elective non-cardiac surgery expected to last more than 1 h under general anesthesia. Exclusion criteria were: (1) patient refusal or inability to provide written informed consent; (2) participation in another clinical trial within the past 30 days; (3) history of schizophrenia, epilepsy, Parkinson’s disease, or myasthenia gravis; (4) severe pre-existing cognitive impairment (Mini-Mental State Examination [MMSE] score (22) < 18); (5) known allergy to benzodiazepines, propofol, or their components; and (6) continuous use of benzodiazepines for more than 1 week within the month prior to surgery.

### Interventions

Upon arrival in the operating room, standard monitoring was established for all participants, including 5-lead electrocardiography, non-invasive blood pressure (NIBP), pulse oximetry (SpO2), and Bispectral Index (BIS) monitoring (Medtronic, United States). The detailed interventions for each group were as follows:

*- Control Group (C group):* Patients in this group received any standard general anesthesia regimen that did not include remimazolam. This typically involved intravenous induction with agents like propofol (1.5–2.5 mg/kg) and maintenance with propofol infusion (4–8 mg/kg⋅h) or volatile anesthetics (e.g., sevoflurane). Opioids and neuromuscular blocking agents were used as in the remimazolam group.

*- Remimazolam Group (R group):* Patients in this group received general anesthesia that included remimazolam tosylate at any stage, either for induction (e.g., 0.1–0.2 mg/kg), for maintenance (e.g., 0.2–1.0 mg/kg⋅h), or both. Opioids (e.g., sufentanil, remifentanil) and neuromuscular blocking agents were used as required.

In both groups, the depth of anesthesia was titrated to maintain a BIS value between 40 and 60. Mechanical ventilation was adjusted to maintain an end-tidal CO2 of 35–45 mmHg. Other aspects of perioperative management, including fluid therapy, hemodynamic support with vasoactive drugs, and postoperative analgesia, were standardized according to institutional protocols to minimize confounding.

### Outcomes

The pre-specified primary outcome was the incidence of POD within the first three postoperative days. Delirium was assessed using the 3-min Confusion Assessment Method, Chinese Version (3D-CAM-CN) (5), by trained researchers at the following timepoints: in the post-anesthesia care unit (PACU) at 10 and 30 min after admission, and subsequently twice daily (between 08:00–10:00 and 18:00–20:00) on postoperative days 1, 2, and 3.

Pre-specified secondary outcomes included: (1) The incidence of emergence agitation in the PACU, evaluated using the Richmond Agitation-Sedation Scale (RASS), with a score of + 1 or higher defined as positive. (2) Postoperative sleep quality, assessed daily for 3 days using a Numerical Rating Scale (NRS; 0 = best possible sleep, 10 = worst possible sleep). (3) Postoperative pain intensity, assessed twice daily for 3 days at rest using the NRS (0 = no pain, 10 = worst imaginable pain). (4) Safety outcomes included the incidence of intraoperative hypotension (defined as requiring vasopressors) at key time points. No changes were made to the trial outcomes after the trial commenced.

### Sample size

The sample size was calculated based on the primary outcome of POD incidence. Based on existing literature and our preliminary observations, we assumed a POD incidence of approximately 50% in the control group for this high-risk frail population (6). We determined that a sample of 295 patients per group would be required to detect a clinically significant 15% absolute risk reduction (i.e., to 35% incidence in the remimazolam group) with 80% power and a two-sided alpha level of 0.05. To account for potential dropouts or incomplete data, we aimed to enroll over 300 patients in each group, for a total target of over 600 patients. No interim analyses or formal stopping guidelines were planned for this observational study.

### Allocation and blinding

This was a non-randomized study. Patient assignment to the Control Group or Remimazolam Group was based on the actual anesthetic administered, as determined by the attending anesthesiologist’s clinical judgment, preference, and patient characteristics before the start of anesthesia. The research team, who were responsible for enrollment and data collection, were not involved in the allocation decision. Blinding (or masking) of outcome assessors was implemented. The research assistants who performed all postoperative delirium, recovery, and pain assessments were not involved in the intraoperative care and were kept unaware of the patient’s group allocation throughout the study period. Due to the distinct physical properties and administration protocols of the anesthetic agents, blinding of patients and attending anesthesiologists to the intervention was not feasible.

### Statistical methods

Data were analyzed using SPSS version 26.0 (IBM Corp., Armonk, NY). For the primary analysis, we compared the incidence of POD between groups using the Chi-square test. For this binary outcome, we calculated both the absolute risk difference and the relative risk with their corresponding 95% confidence intervals (CI). For other outcomes, normally distributed data were compared using the independent *t*-test and expressed as mean ± standard deviation (SD), while non-normally distributed data were compared using the Mann-Whitney U test and expressed as median (interquartile range, IQR). Categorical variables were presented as counts (percentages) and compared using the Chi-square or Fisher’s exact test. To identify independent predictors of POD, a pre-specified multivariate binary logistic regression analysis was performed using the “Enter” method. The model included the treatment group and other clinically relevant variables (age, anesthesia duration, CFS score, and preoperative MMSE score). Results were reported as odds ratios (OR) with 95% CIs. All statistical tests were two-tailed, and a *P* < 0.05 was considered statistically significant. Analysis was performed on an as-treated basis.

## Results

### Participant flow

**FIGURE 1:**
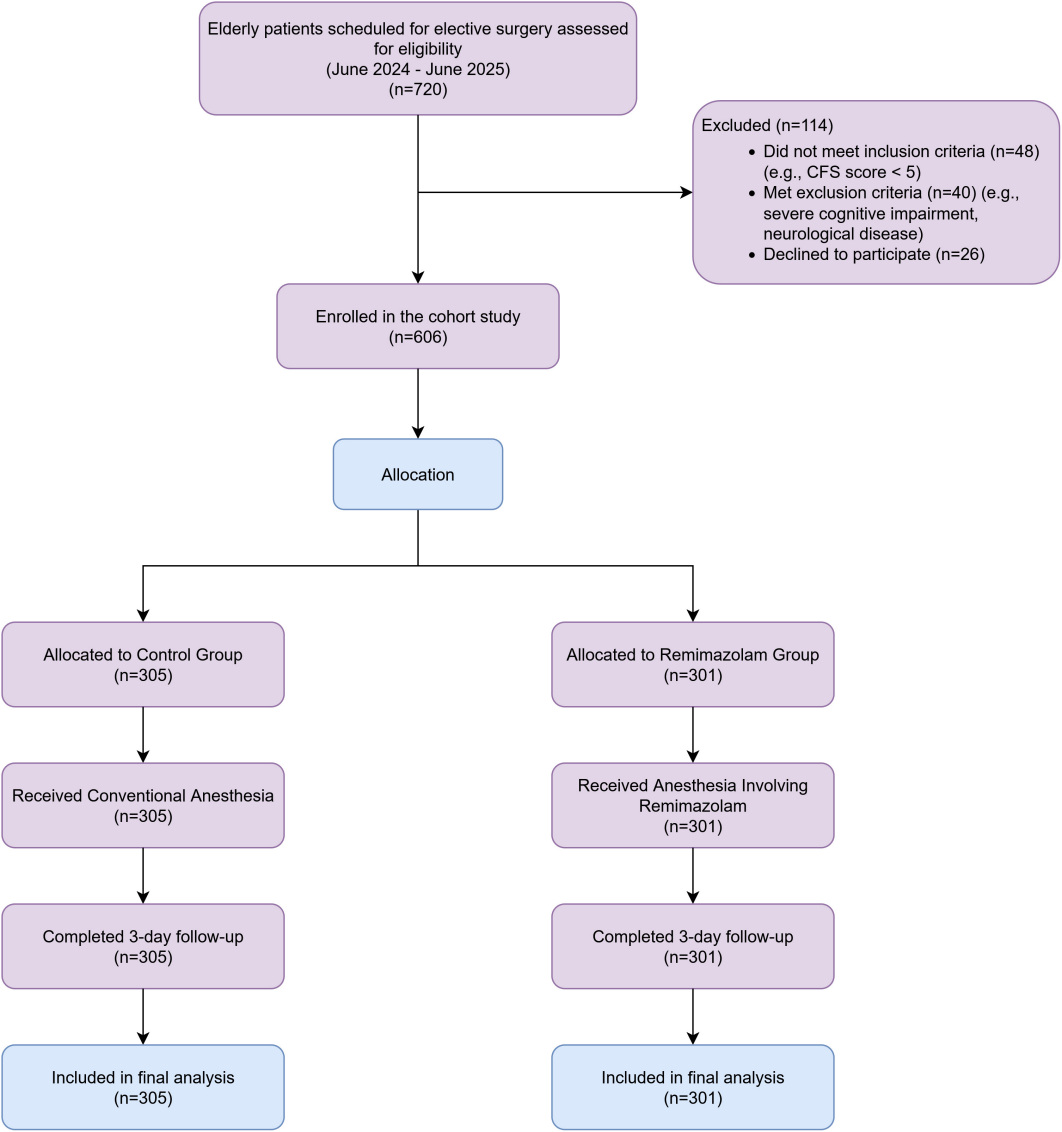
Flow diagram of patient enrollment and allocation.

The flow of participants through the study is detailed in the trial flow diagram (Figure 1). During the recruitment period from June 2024 to June 2025, a total of 720 elderly patients scheduled for elective surgery were assessed for eligibility. Of these, 114 were excluded, most commonly for not meeting the frailty criteria (CFS score < 5, *n* = 48), having pre-existing neurological conditions (*n* = 40), or declining to participate (*n* = 26). Consequently, 606 patients provided informed consent and were enrolled and allocated to a treatment group. All 606 participants (100%) received their assigned anesthetic treatment and completed the full 3-day follow-up. Thus, all enrolled patients were included in the primary outcome analysis. There were no losses to follow-up or exclusions after allocation. The trial was concluded once the target enrollment number was reached.

### Baseline and intraoperative data

The demographic and baseline clinical characteristics for each group are displayed in Table 1. The two groups were well-matched, with no statistically significant differences observed in key variables such as age, gender, BMI, or preoperative MMSE and PSQI scores. Intraoperatively, the median anesthesia duration was significantly shorter in the remimazolam group compared to the control group (170 min vs. 190 min, *P* = 0.019).

**TABLE 1:** Comparison of baseline and intraoperative characteristics

Characteristic	Control group (*n* = 305)	Remimazolam group (*n* = 301)	*P*-value
Gender (male/female), n (%)	159 (52.1)/146 (47.9)	159 (52.8)/142 (47.2)	0.864
Age (years), median (IQR)	67 (52, 94)	69 (50, 97)	0.148
BMI (kg/m^2^), median (IQR)	24.6 (16.1, 41.5)	25.2 (15.2, 43.8)	0.473
CFS Score, median (IQR)	5 (5, 7)	5 (5, 7)	0.022*
MMSE Score, median (IQR)	27 (26, 72)	27 (27, 72)	0.997
PSQI Score, median (IQR)	4 (2, 9)	5 (2, 35)	0.199
Anesthesia duration (min), median (IQR)	190 (60, 845)	170 (35, 840)	**0.019**

*Although the *P*-value for CFS score is 0.022, the medians and ranges are highly overlapping, suggesting minimal clinical difference between groups. Bold value indicates statistical significance (*P* < 0.05).

### Outcomes and estimation

**FIGURE 2:**
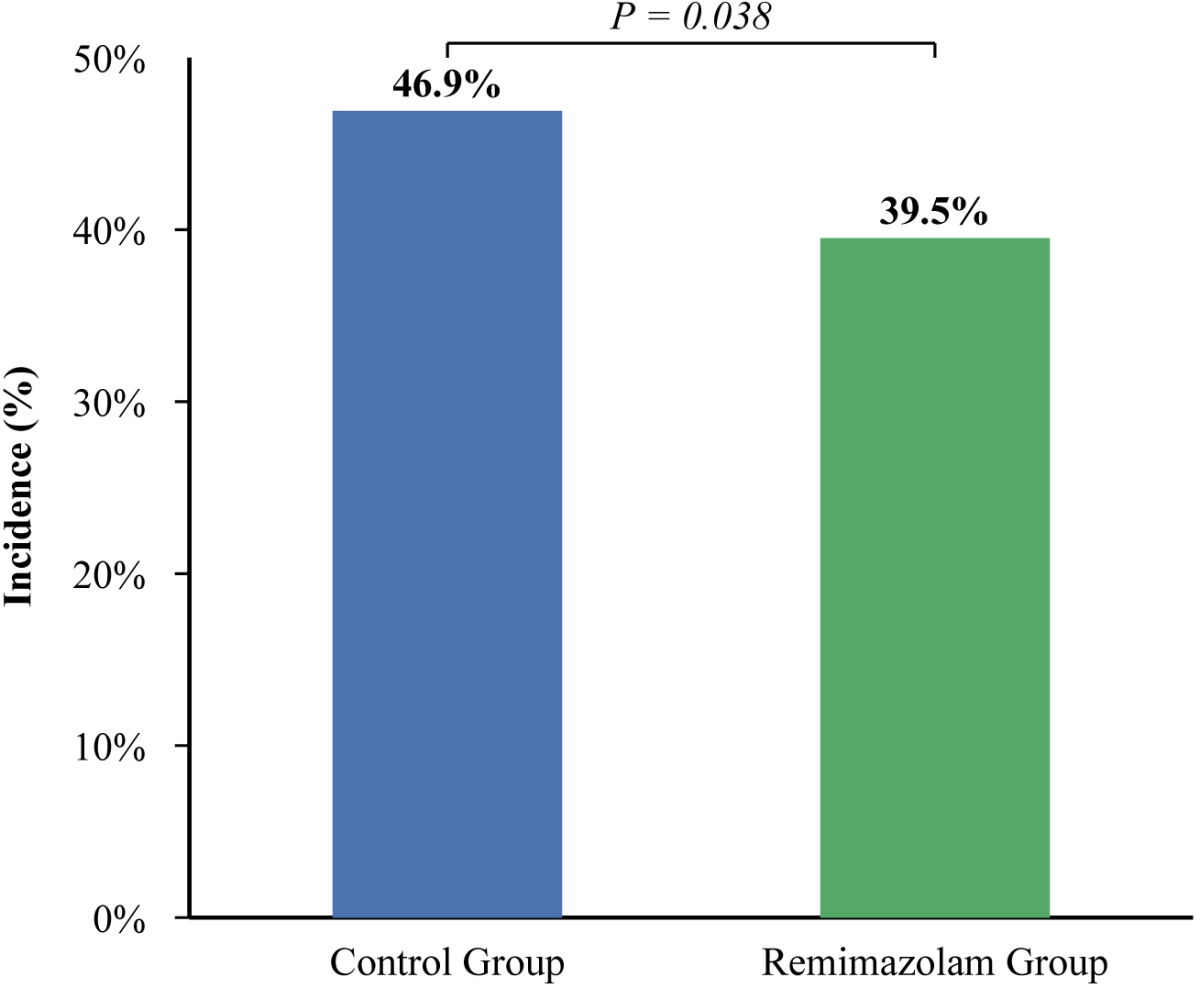
Overall POD incidence.

**FIGURE 3:**
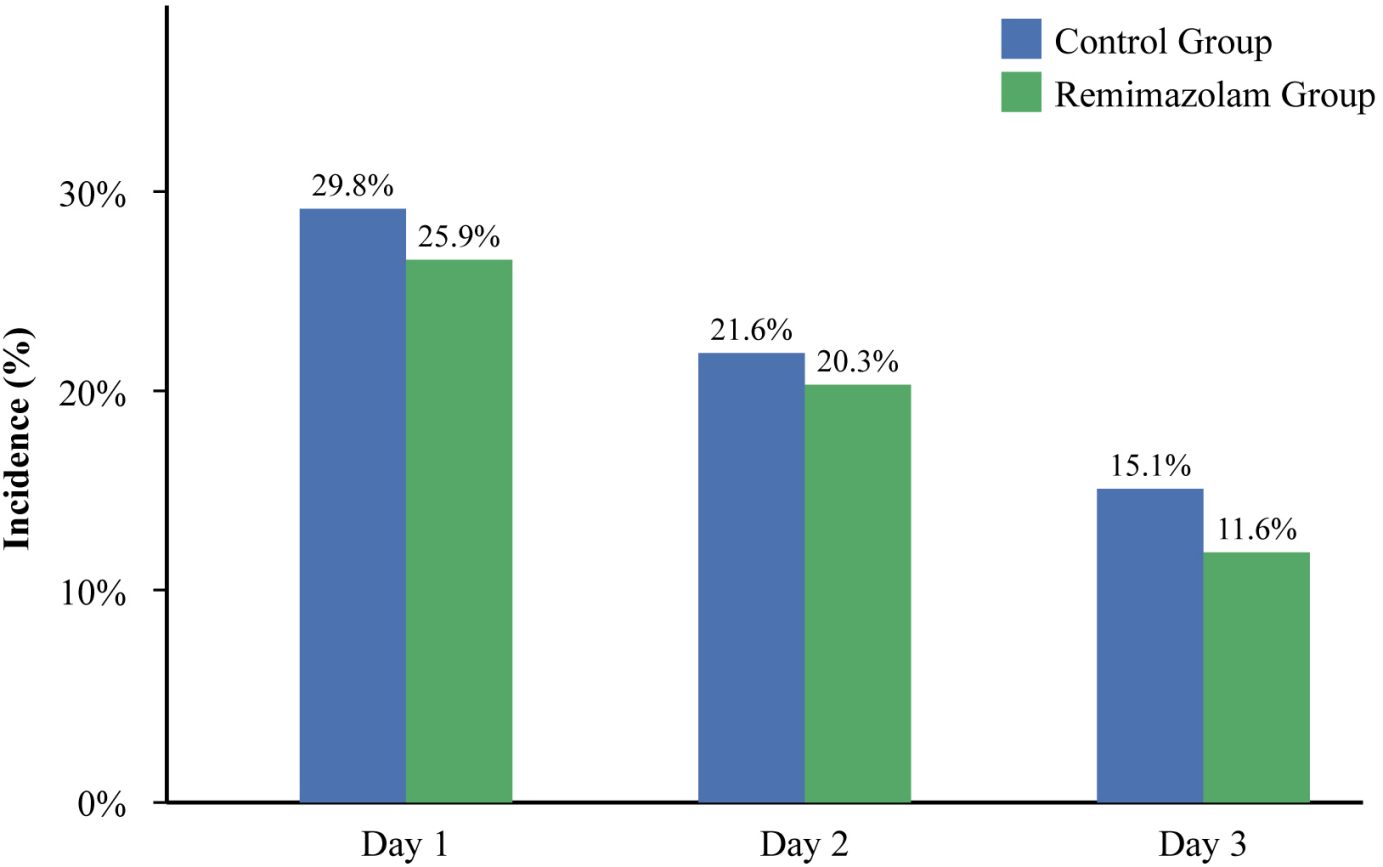
Daily POD incidence on postoperative days 1–3.

For the primary outcome, the overall incidence of POD within the first three postoperative days was 46.9% (143 of 305) in the Control group and 39.5% (119 of 301) in the Remimazolam group (Table 2). This represents a statistically significant absolute risk reduction of 7.4% (95% CI, 0.5–14.3%) and a relative risk of 0.84 (95% CI, 0.71–0.99; *P* = 0.038) (Figure 2). The effect was most pronounced in the immediate postoperative phase, with a significantly lower incidence of delirium at 30 min in the PACU in the R group (18.9% vs. 28.9%, *P* = 0.001). The day-by-day incidence also showed a consistent trend favoring remimazolam (Figure 3).

**TABLE 2:** Comparison of postoperative delirium outcomes.

POD outcome	Control group (*n* = 305)	Remimazolam group (*n* = 301)	*P*-value
Overall POD incidence (Days 1–3), n (%)	143 (46.9)	119 (39.5)	**0.038**
POD in PACU (at 10 min), n (%)	110 (36.1)	101 (33.6)	0.517
POD in PACU (at 30 min), n (%)	88 (28.9)	57 (18.9)	**0.001**
POD duration (days), mean ± SD	2.24 ± 0.73	2.11 ± 0.80	**0.037**

Bold values indicate statistical significance (*P* < 0.05).

**FIGURE 4:**
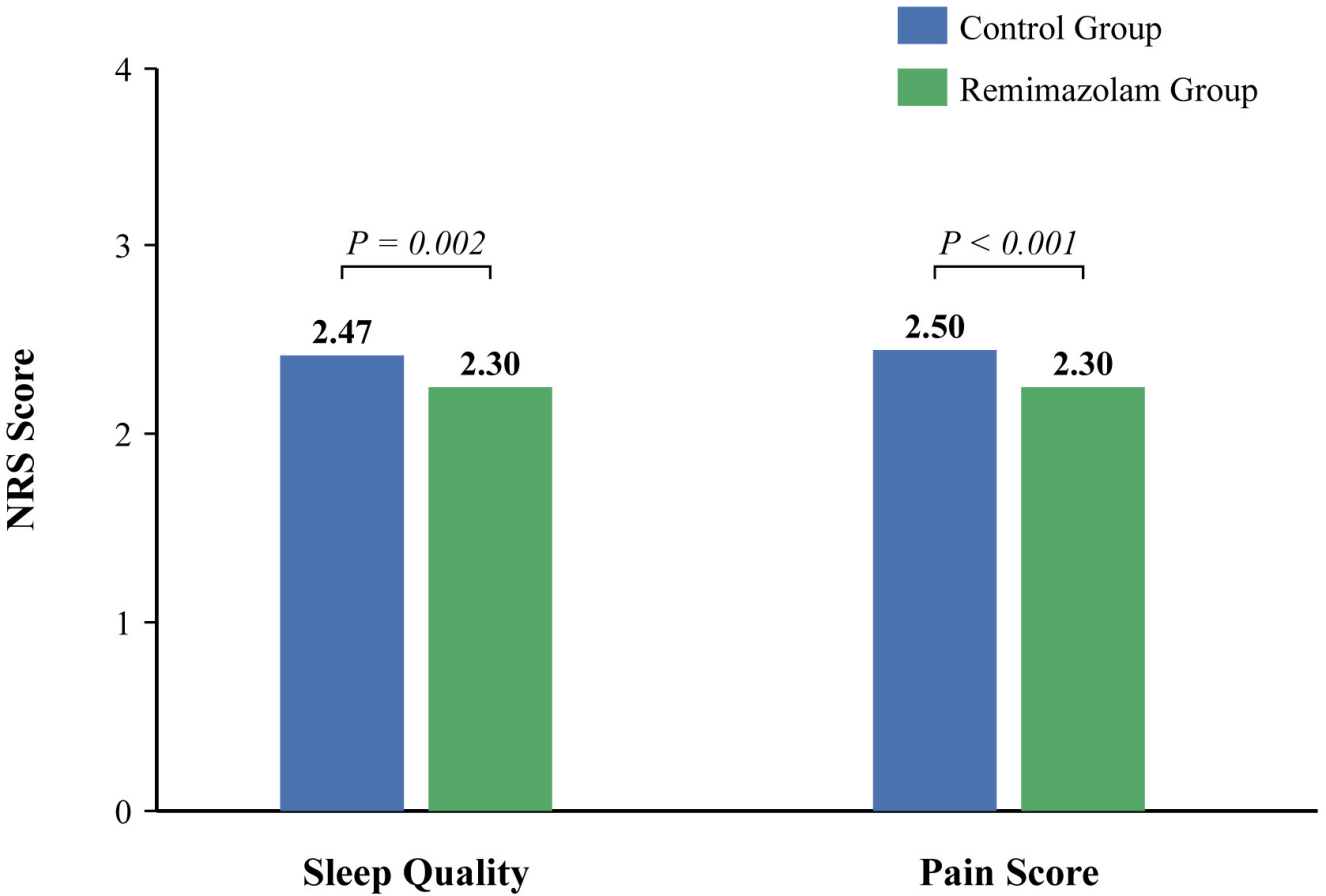
Recovery scores on postoperative day 3.

Regarding secondary recovery outcomes (Table 3; Figure 4), the remimazolam group had a significantly lower incidence of emergence agitation in the PACU (38.9% vs. 41.6%, *P* < 0.05). While sleep quality and pain scores were similar on the first two postoperative days, patients in the R group reported significantly better sleep quality (NRS score: 2.30 ± 0.70 vs. 2.47 ± 0.62, *P* = 0.002) and lower pain scores (NRS score: 2.30 ± 0.62 vs. 2.50 ± 0.64, *P* < 0.001) by the third postoperative day.

**TABLE 3:** Comparison of postoperative recovery quality.

Outcome	Time point	Control group (*n* = 305)	Remimazolam group (*n* = 301)	*P*-value
Emergence agitation (RASS ≥ + 1), n (%)	PACU	127 (41.6)	117 (38.9)	**0.048**
Sleep quality score (NRS), mean ± SD	Day 1	3.61 ± 0.80	3.65 ± 0.91	0.619
	Day 2	2.80 ± 0.72	2.78 ± 0.73	0.581
	**Day 3**	2.47 ± 0.62	2.30 ± 0.70	**0.002**
Pain score (VAS), mean ± SD	Day 1	3.58 ± 0.80	3.56 ± 0.97	0.759
	Day 2	2.75 ± 0.74	2.70 ± 0.69	0.332
	**Day 3**	2.50 ± 0.64	2.30 ± 0.62	**< 0.001**

Bold values indicate statistical significance (*P* < 0.05).

### Harms

Although the incidence of vasopressor use was numerically lower in the R group (20.3% vs. 24.9%), this difference did not reach statistical significance (*P* = 0.160).

### Ancillary analyses

The pre-specified multivariate logistic regression analysis was performed to identify independent predictors of POD after adjusting for potential confounding variables (Table 4). The results confirmed that advanced age (Adjusted OR 1.08 per year, 95% CI 1.05–1.11, *P* < 0.001) and longer anesthesia duration (Adjusted OR 1.02 per 10 min, 95% CI 1.01–1.03, *P* < 0.001) were significant independent risk factors. Critically, after this adjustment, allocation to the remimazolam group remained a significant independent protective factor against the development of POD (Adjusted OR 0.68, 95% CI 0.47–0.98, *P* = 0.041).

**TABLE 4:** Multivariate logistic regression analysis of risk factors for postoperative delirium.

Variable	Odds ratio (OR)	95% confidence interval (CI)	*P*-value
Remimazolam group (vs. control)	**0.68**	**0.47–0.98**	**0.041**
Age (per year increase)	**1.08**	**1.05–1.11**	**< 0.001**
Anesthesia duration (per 10 min increase)	**1.02**	**1.01–1.03**	**< 0.001**
CFS score (per point increase)	1.21	0.98–1.49	0.075
MMSE score (per point increase)	0.97	0.91–1.04	0.415

Bold values indicate statistical significance (*P* < 0.05).

## Discussion

Our study contributes significantly to a growing but conflicting body of literature on the neurocognitive effects of remimazolam. While a recent randomized trial in older orthopedic patients found no significant difference in POD rates between remimazolam and propofol (19), and a meta-analysis suggested no overall superiority for preventing POD (20), our findings align more closely with recent work by Cai et al. (21), who demonstrated a significant reduction in POD in a frail cohort undergoing hip surgery. The primary innovation of our study lies in its large, prospective, real-world cohort design, which specifically targeted a heterogeneous group of frail older patients undergoing various non-cardiac surgeries, rather than focusing on a single surgical specialty. This approach enhances the external validity and generalizability of our findings, suggesting that the benefits of remimazolam may be most apparent in this particularly vulnerable and broadly defined population. Furthermore, our detailed analysis of secondary recovery outcomes provides a more holistic view of the postoperative benefits.

In this large, prospective cohort study of frail elderly patients, we found that the use of remimazolam for general anesthesia, compared to conventional anesthesia, was associated with a significant reduction in the incidence of postoperative delirium. This association persisted after adjusting for key confounders, identifying remimazolam as an independent protective factor. Furthermore, remimazolam use was linked to a more favorable recovery profile, including less emergence agitation, better postoperative sleep, and improved pain control, alongside enhanced intraoperative respiratory stability.

The observed neuroprotective potential of remimazolam is likely multifactorial. First, its unique pharmacokinetic profile—rapid, organ-independent metabolism—minimizes drug accumulation and may reduce the duration and depth of CNS depression, a key trigger for POD (16, 17). This contrasts with propofol, whose clearance can be more variable in older persons. Our findings are in line with a retrospective study by Kaneko et al. (23), who also noted a lower delirium incidence with remimazolam in patients undergoing TAVI. Second, remimazolam contributes to a more stable intraoperative physiological environment. Our data showing a trend towards less severe hypotension aligns with this hypothesis, as maintaining adequate cerebral perfusion is crucial for preventing neuroinflammation (7). It is postulated that stable hemodynamics could mitigate the neuroinflammatory cascade often triggered by surgical stress and hypoperfusion, thereby conferring a neuroprotective effect (24). Third, the benefits extend postoperatively. Pain, sleep disturbance, and agitation are a well-known triad of risk factors for POD (25, 26). By providing a smoother emergence and facilitating better sleep and analgesia, as seen in our Day 3 results, remimazolam may disrupt the vicious cycle where poor recovery exacerbates cognitive dysfunction.

The multivariate analysis confirmed established risk factors like advanced age and long anesthetic exposure (6, 27), which lends validity to our model. The most critical insight, however, is that a modifiable factor—the choice of anesthetic—remains significant. This empowers anesthesiologists to play an active role in neuroprotection.

Our findings also open several avenues for future research that can build upon this work. As suggested by recent frameworks, the development of predictive models for POD in frail older persons is a critical next step. Such models should incorporate not only the choice of anesthetic but also primary disease factors, baseline cognitive status including pre-existing dementia, and overall medication burden, which are known to be significant contributors to delirium risk (28). Furthermore, while our study focused on clinical outcomes, a deeper understanding of the underlying pathophysiology is needed. Future investigations could expand beyond simple inflammatory markers to explore a broader range of biomarkers, including those related to neuroinflammation and oxidative stress, such as the neutrophil-to-lymphocyte ratio (NLR), to elucidate the mechanisms behind remimazolam’s potential neuroprotective effects (29). Our study included a range of non-cardiac surgeries but excluded neurosurgery, which represents a patient population with a particularly high baseline risk of delirium. The potential benefits of remimazolam’s stable hemodynamic profile in neurosurgical anesthesia warrant dedicated investigation. Finally, socioeconomic factors are paramount. Although remimazolam may have a higher acquisition cost, a reduction in POD incidence could translate into shorter hospital stays, reduced complication rates, and lower overall healthcare expenditures. A formal pharmacoeconomic analysis is therefore a crucial and logical next step to assess the overall cost-effectiveness of this anesthetic strategy.

This study has several important limitations that must be acknowledged. First and foremost, the non-randomized design is the most significant source of potential bias. Although we used multivariate regression to adjust for observed baseline differences, unmeasured confounders related to why an anesthesiologist chose one drug over another may still exist (selection bias). Second, the lack of blinding for anesthesiologists could have led to performance bias, where knowledge of the anesthetic might subconsciously influence other aspects of clinical care. Third, as a single-center study conducted in a specific demographic, the generalizability (external validity) of our findings to other institutions with different patient populations or standard practices may be limited. Finally, the analysis of multiple secondary outcomes was not adjusted for multiplicity, and thus these findings should be considered exploratory and hypothesis-generating.

## Conclusion

In conclusion, this comprehensive prospective study demonstrates that in a cohort of frail elderly patients, the use of remimazolam for general anesthesia is a significant independent protective factor against the development of postoperative delirium. This benefit is likely mediated by its favorable pharmacokinetic profile, its ability to maintain greater intraoperative physiological stability, and its positive impact on the overall quality of postoperative recovery. While randomized evidence is needed, our findings strongly suggest that the choice of remimazolam represents a valuable, modifiable strategy for optimizing perioperative care and improving neurological outcomes in this vulnerable population. Future research should focus on large-scale, multicenter randomized controlled trials to confirm these findings and establish a definitive causal relationship.

## Data Availability

The original contributions presented in the study are included in the article/supplementary material, further inquiries can be directed to the corresponding authors.
